# Risk Factors and Genetic Biomarkers of Multiple Primary Cancers in Esophageal Cancer Patients

**DOI:** 10.3389/fonc.2020.585621

**Published:** 2021-01-22

**Authors:** Pei-Wen Yang, Mei-Chun Lin, Pei-Ming Huang, Cheng-Ping Wang, Tseng-Cheng Chen, Chun-Nan Chen, Mong-Hsun Tsai, Jason Chia-Hsien Cheng, Eric Y. Chuang, Min-Shu Hsieh, Pei-Jen Lou, Jang-Ming Lee

**Affiliations:** ^1^ Department of Surgery, National Taiwan University Hospital and National Taiwan University College of Medicine, Taipei, Taiwan; ^2^ Department of Surgery, National Taiwan University Cancer Center, Taipei, Taiwan; ^3^ Department of Otolaryngology, National Taiwan University Hospital and College of Medicine, Taipei, Taiwan; ^4^ Graduate Institute of Biomedical Electronics and Bioinformatics, National Taiwan University, Taipei, Taiwan; ^5^ Department of Oncology, National Taiwan University Hospital and National Taiwan University College of Medicine, Taipei, Taiwan; ^6^ Biomedical Technology and Device Research Laboratories, Industrial Technology Research Institute, Hsinchu, Taiwan; ^7^ Department of Pathology, National Taiwan University Hospital and National Taiwan University College of Medicine, Taipei, Taiwan

**Keywords:** esophageal cancer, multiple primary cancer, second primary cancer, single-nucleotide polymorphism, head and neck cancer

## Abstract

Esophageal cancer (EC) is a deadly cancer that frequently develops multiple primary cancers (MPCs). However, the risk biomarkers of MPC in EC have hardly been investigated. We retrospectively enrolled 920 subjects with primary EC and analyzed the possible risk factors as well as MPC single-nucleotide polymorphisms (SNPs) from blood DNA. A total of 184 subjects (20.0%) were confirmed to have MPC, 59 (32.8%) had synchronous MPC, and 128 (69.6%) had head and neck cancer. Elderly EC patients have an increased risk of having gastrointestinal cancer (Odds ratio, OR[95% CI]=6.70 [1.49–30.19], *p*=0.013) and a reduced risk of developing HNC (OR[95% CI]=0.44 [0.24–0.81], *p*=0.008). MPC risk was also associated with betel nut chewing (OR[95% CI]=1.63, 1.14–2.32], *p*=0.008), the A allele of ALDH2:rs671 (*p*=0.074 and 0.030 for GA and AA, respectively), the CC genotype in CISH:rs2239751 (OR[95% CI]=1.99 [1.2–3.32], *p*=0.008), and the G allele of ERCC5:rs17655 (*p*=0.001 and 0.090 for GC and CC, respectively). ADH1B:rs1229984 also correlated with MPC risk (*p*=0.117). Patients carrying four risk SNPs had a 40-fold risk of MPC (OR[95% CI]=40.25 [6.77–239.50], *p*<0.001) and a 12.57-fold risk of developing second primary cancer after EC (OR[95% CI]=12.57 [1.14–138.8], *p*=0.039) compared to those without any risk SNPs. In conclusion, hereditary variations in ALDH2, CISH, ERCC5, and ADH1B have great potential in predicting the incidence of MPC in EC patients. An extensive cancer screening program during clinical follow-up would be beneficial for patients with high MPC susceptibility.

## Introduction

Esophageal cancer (EC) is a deadly disease. Primary EC most often presents either as esophageal squamous cell carcinoma or adenocarcinoma (1, 2). Accounting for over 90% of the disease worldwide, esophageal squamous cell carcinoma is the major cell type of primary EC and is highly correlated with environmental factors (3, 4). Moreover, it is also highly correlated with unfavorable habits, such as tobacco smoking, alcohol drinking, and betel nut chewing (3, 5). Compared to patients without EC, studies indicate that EC patients have a more than 12-fold risk of developing mouth/pharynx cancer (6) and about a 4-fold risk of stomach cancer (6).

Multiple primary cancers (MPCs) are defined as more than one (synchronous or metachronous) primary cancer in the same individual (7). The frequency of multiple primaries for cancer patients is reported to be in the range of 2–17% (7). A cancer patient may develop multiple primary tumors due to several epidemiological factors, such as genetics, family history, hormonal factors, prior cancer treatment, lifestyle factors, and environmental influences (7). There is a 5–10% risk of MPC with an inherited genetic mutation (8). Systematic biomarker studies for MPCs are lacking, especially in patients with EC. Here, we investigated the association between candidate single-nucleotide polymorphisms (SNPs) and MPC in EC patients. These candidates include the SNPs at the aldehyde dehydrogenase 2 family member (*ALDH2*), alcohol dehydrogenase 1B (*ADH1B*), cytochrome P450 family 1 subfamily A member 1 (*CYP1A1*), glutathione S-transferase pi 1 (*GSTP1*), cytochrome c oxidase subunit 2 (COX2, encoded by *PTGS2* gene), and ERCC excision repair 2 (*ERCC5*).

Alcohol dehydrogenase (ADH) and aldehyde dehydrogenase (ALDH) are key NAD-dependent enzymes involved in alcohol metabolism (9). ADH1B and ALDH2 are the major enzymes that convert alcohol to acetate in humans (10). The ADH1B SNPs rs1229984 and ALDH2:rs671(G>A, Glu487Lys) are reportedly correlated with the risk of alcohol-related cancers, including hypopharyngeal cancer and EC (11–16). Additionally, COX2:rs20417 and CYP1A1:rs1048943 are known to correlate with the risk of both oral cancer and EC (17–19). SNPs involved in nucleotide excision repair, such as ERCC5:rs17655, have also been found to be associated with the incidence of laryngeal cancer and EC (20, 21). Finally, GSTP1 rs1695 is also an SNP for the risk of EC and other cancers, such as breast cancer (22, 23).

In the current study, we investigated the risk factors and potential genetic biomarkers for MPC in patients with EC.

## Materials and Methods

### Study Population and Data Collection

**Figure 1:**
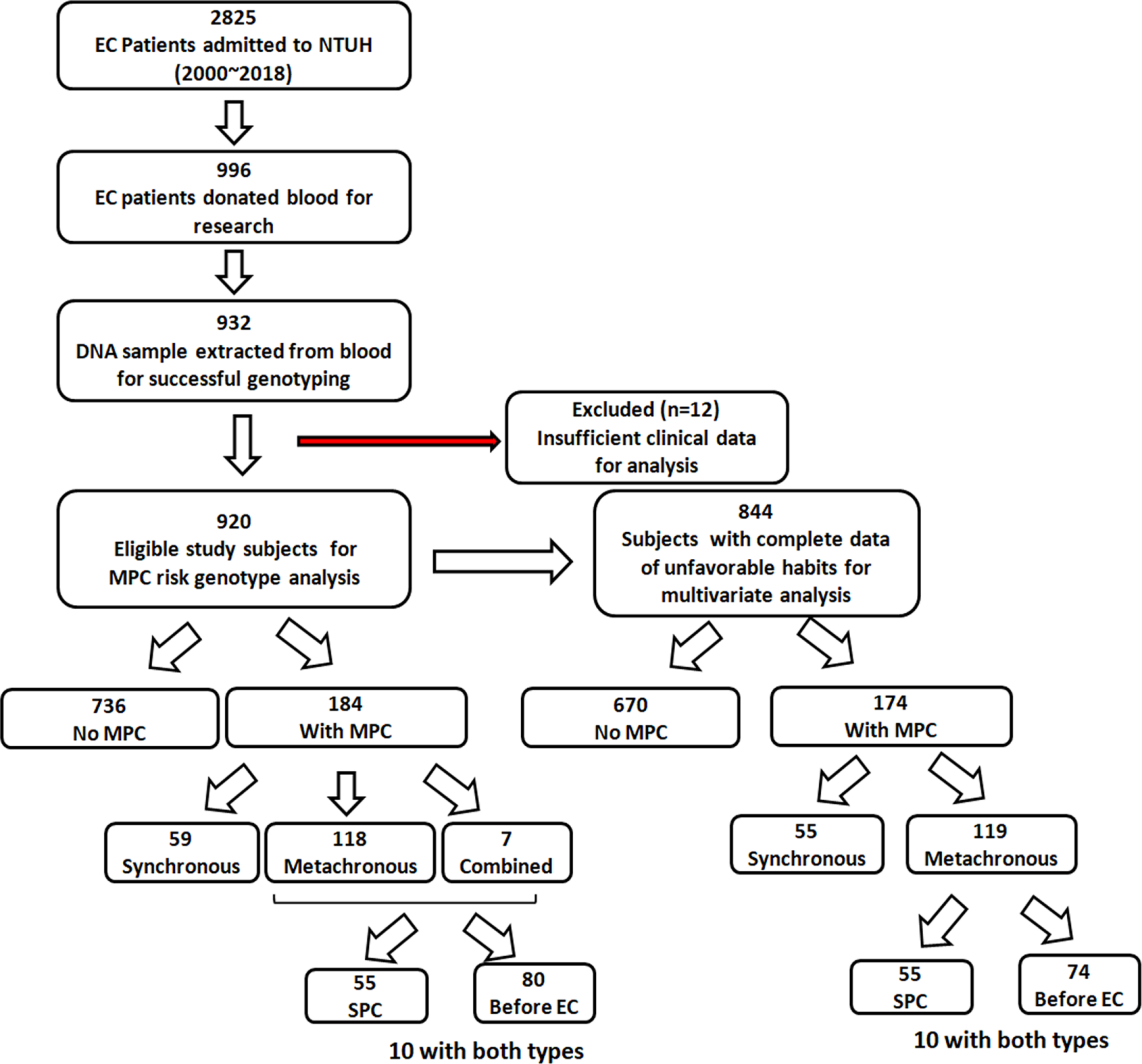
Flow chart showing the patient recruitment process and the number of eligible study subjects and subgroups.

This study was performed retrospectively and was approved by the ethical committee of the National Taiwan University Hospital (NTUH, 201803015RIND). A total of 920 EC patients with or without MPC were retrospectively enrolled in the National Taiwan University Hospital (NTUH) during the study period (Jan. 2000 to Dec. 2018). The inclusion criteria were patients diagnosed with primary EC. Pregnant patients, pediatric patients, and those unable to give informed consent or blood samples were excluded. **Figure 1** shows the study flowchart. In total, 2825 patients with primary EC were admitted to NTUH between 2000 and 2018. Among them, 996 patients donated blood for research, and 932 DNA samples were successfully extracted from the blood samples for genotyping. Twelve patients were excluded owing to insufficient clinical data for analysis. The 920 eligible study subjects included 736 patients without MPC during their follow-up and 184 patients who were diagnosed with MPC.

Data concerning unfavorable habits, including cigarette smoking, alcohol drinking, and betel nut chewing, were collected from each patient during their clinic visit, and confirmed by nursing documentation; the information was sometimes also verified by the patient or their close family member *via* the telephone. Any ambiguous or vague information regarding the habits of the patients was considered as “missing data.” Every user was considered to have a history of unfavorable habits. Data concerning smoking, drinking, and betel nut chewing were missing in 56, 54, and 76 subjects, respectively. A total of 844 patients were thus included in the multivariate analysis, which adjusted for age, gender, tumor site, history, and these unfavorable habits (**Figure 1**).

Basic demographics, unfavorable habits, and time of primary cancer onset were obtained from the Tumor Registry of NTUH and/or medical chart review. EC treatment included surgery, chemotherapy, and radiotherapy. Neoadjuvant concurrent chemoradiation therapy was administered to patients with advanced TNM stages (T3N0 or T1-3N+) (24) diagnosed using endoscopic ultrasound or computed tomography before surgery (25, 26).

The Warren and Gates criteria for second primary cancer (SPC) were used to defined MPC (27). MPCs were classified as synchronous if the different primary cancers were diagnosed simultaneously or within 6 months of primary EC diagnosis. If the interval between the date of EC diagnosis and that of another cancer was >6 months, the MPC was considered metachronous. Patients who had both synchronous and metachronous cancer were classified as having combined synchronous and metachronous cancer (28).

The organ sites of the MPCs in EC patients were listed in **Table 1**. The MPC type was further classified according to anatomical location. Head and neck cancer (HNC) includes oral (oral cavity and tongue), hypopharyngeal, nasopharyngeal, oropharyngeal, laryngeal, pharyngeal, tonsil, and thyroid cancers. Gastrointestinal cancer includes gastric, liver, colon, rectal, pancreatic, cecal, and gallbladder cancers. Thoracic cancer includes lung/bronchus and tracheal cancer. Other types of SPC include bladder, breast, prostate cancer, renal, bone, skin, eye cancers, and lymphoma.

**Table 1:** The organ sites of MPC in EC patients.

MPC by organ sites	Case number
Hypopharynx	57
Oropharynx	16
Nasopharynx	1
Oral cavity	24
Tongue	23
Tonsil	6
Larynx	12
Pharynx	1
Thyroid	7
Stomach	14
Liver	8
Colon	6
Pancreas	1
Cecum	1
Gallbladder	1
Rectum	3
Kidney	1
Lung	18
Trachea	1
Bronchus	1
Bladder	3
Lymphoid	1
Breast	1
Skin	1
Bone	1
Prostate	2
Eye	1

### DNA Extraction

Buffy coat samples were isolated from 10 mL of whole blood, which was obtained from patients before cancer treatment and stored in a -80°C freezer. Genomic DNA was extracted from 200 μL of the buffy coat containing peripheral blood mononuclear cells using the QIAamp DNA mini kit (Qiagen, Hamburg Germany) according to the manufacturer’s instructions. The DNA was dissolved in double-distilled water and stored in a -20°C freezer.

### SNP Genotyping

The genotyping of the candidate SNPs (ADH1B:rs1229984, ALDH2:rs671, COX2:rs20417, CYP1A1:rs1048943, ERCC5:rs17655, GSTP1:rs1695, SOCS1:rs243324, SOCS1:rs33932899, and CISH:rs2239751) was performed using the Sequenom MassARRAY platform and iPLEX gold chemistry and was analyzed using MassARRAY TYPER 4.0 software following the manufacturer’s instructions (Sequenom, San Diego, CA) as previously described (29). Data integrity and accuracy were confirmed through repeated measures. Genotyping for rs671, rs243324, and rs2239751 was complete. There were six, five, three, four, four, and two patients with unconfirmed data for rs1229984, rs20417, rs1048943, ERCC5:rs17655, GSTP1:rs1695, and SOCS1:rs33932899, respectively.

The genotype distribution data supporting the findings of this study are publicly available. These data can be found here: https://doi.org/10.2217/14622416.9.2.235 (Taiwan population) and https://doi.org/10.1101/531210 (East Asia population).

### Statistical Analysis

All statistical analyses were conducted using SPSS 17.0 (SPSS Institute, Chicago, IL, USA). Patient characteristics and genotype distribution among the subgroups with or without MPC were compared using Pearson’s chi-square test or Fisher’s exact test. The odds ratios (ORs) for MPC of patients carrying risk genotypes or factors adjusted for potential covariates were analyzed using binary logistic regression. A *p*-value of less than 0.05 was considered statistically significant. Receiver operating characteristic (ROC) analysis was used to evaluate the diagnostic performance of the risk genotypes for MPC. The area under the curve (AUC) or the ROC curve was applied to evaluate the discriminatory capability of the risk genotypes for patients with SPC after EC. Generally, an AUC of 0.7–0.8 is considered acceptable, and 0.8–0.9 is considered excellent (30).

## Results

Of the 920 EC patients, 20.0% (N=184) were confirmed to have MPC (**Table 2**). Among these 920 patients, 32.8% (N=59) had synchronous MPC, 64.1% (N=118) developed metachronous MPC, and 3.8% (N=7) had combined synchronous and metachronous MPC. Within the metachronous and combined MPC groups (N=125), 55 patients developed SPC after EC, and 80 patients had other malignant tumors before being diagnosed with EC (**Figure 1**). Regarding the site distribution of MPC in EC patients, the site most frequently affected was the head and neck (N=128, 69.6%). In addition, 21.1% (N=39) and 10.9% (N=20) of the MPC patients were diagnosed with cancer of the digestive system and thoracic cavity, respectively. A total of 28 patients had multiple MPC with cancer develop from two to five organs.

**Table 2:** Patient Characteristics.

Number	Total EC patients			*p*-value	With MPC				*p*-value
	Total	No MPC	with MPC		Total	Synchronous	Metachronous	Combined	
	920 (100.0)	736 (80.0)	184 (20.0)		184 (100.0)	59 (32.8)	118 (64.1)	7 (3.8)	
**Age (years)**				0.373					0.596
<50	194 (21.1)	155 (79.9)	39 (20.1)		39 (21.2)	13 (33.3)	25 (64.1)	1 (2.6)	
50-65	454 (49.3)	356 (78.4)	98 (21.6)		98 (53.3)	27 (27.6)	66 (67.3)	5 (5.1)	
>65	272 (29.6)	225 (82.7)	47 (17.3)		47 (25.5)	19 (40.4)	27 (57.4)	1 (2.1)	
**Gender**									
Female	68 (7.4)	61 (89.7)	7 (10.3)	**0.038**	7 (3.8)	1 (14.3)	6 (85.7)	0 (0)	0.565
Male	852 (92.6)	675 (79.2)	177 (20.8)		177 (96.2)	58 (32.8)	112 (63.3)	7 (4.0)	
**Tumor Site**				0.200					0.270
Upper	168 (18.3)	142 (84.5)	26 (15.5)		26 (14.1)	10 (38.5)	13 (50.0)	3 (11.5)	
Middle	319 (34.7)	251 (78.7)	68 (21.3)		68 (37.0)	24 (35.3)	42 (61.8)	2 (2.9)	
Lower	270 (29.3)	219 (81.1)	51 (18.9)		51 (27.7)	15 (29.4)	36 (70.6)	0 (0)	
Upper-middle	37 (4.0)	30 (81.1)	7 (18.9)		7 (3.8)	3 (42.9)	4 (57.1)	0 (0)	
Lower-middle	117 (12.7)	89 (76.1)	28 (23.9)		28 (15.2)	7 (25.0)	19 (67.9)	2 (7.1)	
Multiple	9 (1.0)	5 (55.6)	4 (44.4)		4 (2.2)	0 (0)	4 (100.0)	0 (0)	
**Cell type**				0.48					0.744
ESCC	894 (97.2)	714 (79.9)	180 (20.1)		180 (97.8)	59 (32.8)	114 (63.3)	7 (3.9)	
EADC	23 (2.5)	20 (87.0)	3 (13.0)		3 (1.6)	0 (0)	3 (100.0)	0 (0)	
Others	3 (0.3)	2 (66.7)	1 (33.3)		1 (0.5)	0 (0)	1 (100.0)	0 (0)	
**CRT**				0.407					0.300
No	208 (22.7)	167 (80.3)	41 (19.7)		41 (22.3)	8 (19.5)	31 (75.6)	2 (4.9)	
CCRT	677 (73.8)	537 (79.3)	140 (20.7)		140 (76.1)	50 (35.7)	85 (60.7)	5 (3.6)	
CT only	20 (2.2)	19 (95.0)	1 (5.0)		1 (0.5)	0 (0)	1 (100.0)	0 (0)	
RT only	12 (1.3)	10 (83.3)	2 (16.7)		2 (1.1)	1 (50.0)	1 (50.0)	0 (0)	
**Cigarette-smoking**				**0.03**					0.652
No	131 (15.2)	113 (86.3)	18 (13.7)		18 (10.0)	5 (27.8)	12 (66.7)	1 (5.6)	
Yes	733 (84.8)	571 (77.9)	162 (22.1)		162 (90.0)	53 (32.7)	103 (63.6)	6 (3.7)	
**Alcohol-drinking**				**0.006**					0.366
No	163 (18.8)	142 (87.1)	21 (12.9)		21 (11.7)	4 (19.0)	16 (76.2)	1 (4.8)	
Yes	703 (81.2)	545 (77.5)	158 (22.5)		158 (88.3)	53 (33.5)	99 (62.7)	6 (3.8)	
**Betel-chewing**				**0.001**					**0.028**
No	545 (64.6)	452 (82.9)	93 (17.1)		93 (53.4)	23 (24.7)	68 (73.1)	2 (2.2)	
Yes	299 (35.4)	218 (72.9)	81 (27.1)		81 (46.6)	32 (39.5)	44 (54.3)	5 (5.2)	

EC, esophageal cancer; MPC, multiple primary cancer. Bold values indicate statistical significance with a p value less than 0.05.

MPC incidence was significantly higher among males (20.8% vs. 10.3%, male vs. female, *p*=0.038, **Table 2**). As expected, the unfavorable habits of cigarette smoking, alcohol drinking, and betel nut chewing were all correlated with increased MPC incidence (*p*=0.030, 0.006, and 0.001, respectively, **Table 2**). Notably, patients with a history of betel chewing developed synchronous MPC more frequently than those without (39.5% vs. 24.7%, *p*=0.028, **Table 2**). We further analyzed the risk factors of individual MPC types using logistic regression adjusted for other variables. Among these unfavorable habits, betel chewing was found to be a significant risk factor for the incidence of MPC and HNC even after adjusting for other covariates (*p*=0.008 and *p*=0.038, respectively, **Table 3**), especially for synchronous MPC (OR[95% CI]=2.43 [1.36–4.35], *p*=0.003, **Table 4**). Moreover, elderly EC patients were found to have a reduced risk of HNC (OR[95% CI]=0.44 [0.24–0.81], *p*=0.008, **Table 3**). However, they had a 6-fold increased risk of having gastrointestinal cancer (OR[95% CI]=6.70 [1.49–30.19], **Table 3**). Having multiple superficial ECs was also significantly correlated with a higher risk of MPC and HNC (OR[95% CI]=5.38 [1.30–22.25], *p*=0.020 for MPC; 6.78 [1.60–28.75], *p*=0.009 for head and neck MPC, **Table 3**).

**Table 3:** Risk factors for MPC analyzed by multivariate logistic regression.

Variables	Total	Risk for MPC (N = 174)		Risk for HNC (N = 122)		Risk for GI Ca. (N = 37)		Risk for thoracic Ca. (N = 19)	
		adjusted OR (95% CI)	*p-value	adjusted OR (95% CI)	*p-value	adjusted OR (95% CI)	*p-value	adjusted OR (95% CI)	*p-value
**Age (years)**									
<50	175	1		1		1		1	
50-65	425	0.93 (0.60-1.43)	0.740	0.77 (0.48-1.23)	0.270	4.56 (1.05-19.89)	**0.043**	0.52 (0.14-2.00)	0.343
>65	244	0.91 (0.55-1.51)	0.717	0.44 (0.24-0.81)	**0.008**	6.70 (1.49-30.19)	**0.013**	2.26 (0.67-7.68)	0.190
**Gender**									
Female	63	1		1		1		1	
Male	781	1.93 (0.76-4.88)	0.167	1.34 (0.47-3.77)	0.586	–	0.997	1.08 (0.11-10.38)	0.947
**Tumor Site**									
Upper	154	1		1		1		1	
Middle	296	1.52 (0.90-2.54)	0.115	1.50 (0.83-2.71)	0.184	0.86 (0.31-2.39)	0.770	1.20 (0.30-4.77)	0.799
Lower	246	1.30 (0.76-2.24)	0.342	1.32 (0.70-2.47)	0.389	1.12 (0.40-3.13)	0.830	0.85 (0.18-3.93)	0.834
Upper-middle	34	1.21 (0.47-3.12)	0.697	0.93 (0.29-3.00)	0.905	0.70 (0.08-6.10)	0.744	2.62 (0.41-16.81)	0.31
Lower-middle	105	1.48 (0.78-2.82)	0.229	1.38 (0.66-2.88)	0.396	1.51 (0.46-4.94)	0.493	1.73 (0.33-8.99)	0.515
Multiple sites	9	5.38 (1.30-22.25)	**0.020**	6.78 (1.60-28.75)	**0.009**	–	0.999	–	0.999
**Cell type**									
ESCC	820	1		1		1		1	
EADC	22	0.77 (0.22-2.74)	0.685	0.39 (0.05-3.02)	0.367	3.46 (0.87-13.81)	0.079	–	0.998
Others	2	–	0.999	–	0.999	–	1.000	–	1.000
**Cigarette-smoking**									
No	130	1		1		1		1	
Yes	714	1.02 (0.54-1.95)	0.942	1.10 (0.50-2.37)	0.831	1.37 (0.40-4.69)	0.612	1.08 (0.18-6.69)	0.932
**Alcohol-drinking**									
No	163	1		1		1		1	
Yes	681	1.45 (0.80-2.62)	0.221	1.66 (0.79-3.47)	0.179	0.78 (0.29-2.11)	0.623	2.23 (0.37-13.67)	0.385
**Betel-chewing**									
No	545	1		1		1		1	
Yes	299	1.63 (1.14-2.32)	**0.008**	1.54 (1.02-2.32)	**0.038**	1.52 (0.75-3.07)	0.249	1.32 (0.50-3.49)	0.575

MPC, multiple primary cancer; HNC, head and neck cancer; GI, gastrointestinal; Ca, cancer; ESCC, esophageal squamous cell carcinoma; EADC, esohgeal adenocarcinoma. Bold values indicate statistical significance with a p value less than 0.05.

**Table 4:** Risk factors for synchronous and metachronous MPC analyzed by multivariate logistic regression.

Variables	Total	Risk for synchronous MPC (N=55)		Risk for metachronous MPC (SPC, N=55)		Risk for metachronous MPC (before EC, N=74)	
		adjusted OR (95% CI)	*p-value	adjusted OR (95% CI)	*p-value	adjusted OR (95% CI)	*p-value
**Age (years)**							
<50	175	1		1		1	
50-65	425	0.77 (0.38-1.56)	0.464	0.80 (0.41-1.53)	0.494	1.31 (0.69-2.50)	0.408
>65	244	1.34 (0.62-2.90)	0.460	0.45 (0.19-1.05)	0.064	1.09 (0.51-2.32)	0.827
**Gender**							
Female	63	1		1		1	
Male	781	2.56 (0.31-20.97)	0.380	1.23 (0.33-4.58)	0.762	2.59 (0.55-12.15)	0.228
**Tumor Site**							
Upper	154	1		1		1	
Middle	296	1.32 (0.59-2.97)	0.502	1.78 (0.77-4.08)	0.176	0.85 (0.41-1.74)	0.650
Lower	246	1.00 (0.42-2.41)	0.996	1.09 (0.44-2.72)	0.857	1.06 (0.51-2.21)	0.879
Upper-middle	34	1.23 (0.31-4.90)	0.771	1.14 (0.23-5.71)	0.872	0.99 (0.26-3.72)	0.986
Lower-middle	105	0.98 (0.33-2.91)	0.977	1.27 (0.44-3.65)	0.659	1.31 (0.56-3.07)	0.542
Multiple	9	–	0.999	–	0.999	10.49 (2.34-47.09)	**0.002**
**Cell type**							
ESCC	820	1		1		1	
EADC	22	–	0.998	1.94 (0.40-9.39)	0.411	0.57 (0.07-4.50)	0.596
Others	2	–	0.999	–	0.999	–	0.999
**Cigarette-smoking**							
No	130	1		1		1	
Yes	714	0.76 (0.26-2.25)	0.621	0.82 (0.31-2.15)	0.684	1.48 (0.54-2.84)	0.442
**Alcohol-drinking**							
No	163	1		1		1	
Yes	681	0.75 (0.26-2.25)	0.141	1.08 (0.43-2.71)	0.873	1.22 (0.52-2.84)	0.651
**Betel-chewing**							
No	545	1		1		1	
Yes	299	2.43 (1.36-4.35)	**0.003**	1.38 (0.77-2.46)	0.275	1.31 (0.79-2.18)	0.298

*adjusted for other variables; MPC, multiple primary cancer; SPC, Second primary cancer. Bold values indicate statistical significance with a p value less than 0.05.

We preliminarily analyzed the association between esophageal cancer MPC and 58 single nucleotide polymorphisms (SNPs) that are related to carcinogenesis. The SNPs were determined using our pre-existing database of a smaller cohort (N=500, data not shown). These SNPs included 16 SNPs involved in growth factors and receptors, 10 SNPs related to microRNA functions, eight SNPs associated with inflammation, four SNPs of the genes of the nucleotide excision repair (NER) pathway, and 20 SNPs of the genes of the suppressor of cytokine signaling (SOCS) family. The preliminary results revealed that three SNPs of the suppressor of cytokine signaling (SOCS) family of genes, including CISH:rs2239751, SOCS1:rs33932899, and SOCS1:rs243324, showed a tendency to correlate with increased MPC risk in EC. In addition to the three SNPs, we added six candidate SNPs for MPC analysis, including ADH1B:rs1229984, ALDH2:rs671 (11–16), COX2:rs20417, CYP1A1:rs1048943 (17–19), ERCC5:rs17655 (20, 21), and GSTP1:rs1695 (22, 23). Furthermore, we analyzed the feasibility of using these nine candidate SNPs as biomarkers in predicting the incidence of EC and MPC.

We first compared genotype distributions of the nine candidate genes among normal populations of Taiwan and East Asia, as well as the EC subjects enrolled in this study. The genotype distributions of the Taiwan and East Asia populations were not significantly different from all SNPs (**Table 5**). On the other hand, there were highly significant differences between the EC patients and the normal Taiwanese population in terms of distributions of both alcohol-related SNPs (ADH1B:rs1229984 and ALDH2:rs671, *p*<0.001). In ADH1B:rs1229984, the homologous variant CC was significantly more prevalent in the EC patients than in the normal Taiwanese population; by contrast, TT was significantly less common (23.5% vs. 7.4% for CC; 39.1% vs. 52.2% for TT, *p*<0.001, **Table 5**). Individuals carrying the CC genotype had a 4.30-fold increased risk for EC (CC/TT, crude OR[95% CI]=4.30 [3.32–5.58], *p*<0.001, **Table 6**).

**Table 5:** Genotype distribution of esophageal cancer patients.

SNP/Gene	Genotype	#EC	*Taiwan (TW)	&East Asia (EA)	P-value (EC vs. Taiwan)	P-value (TW vs. EA)
**rs1048943**	**TT**	508 (55.4)	859 (56.6)	5649 (56.7)	0.919	0.999
***CYP1A1***	**CT**	348 (37.9)	570 (37.6)	3731 (37.4)		
	**CC**	61 (6.7)	88 (5.8)	590 (5.9)		
**rs1229984**	**TT**	357 (39.1)	789 (52.2)	5424 (54.4)	**<0.001**	0.905
***ADH1B***	**TC**	339 (37.1)	611 (40.4)	3864 (38.7)		
	**CC**	218 (23.8)	112 (7.4)	684 (6.9)		
**rs1695**	**AA**	596 (65.1)	1026 (67.8)	6650 (68.2)	0.711	0.955
***GSTP1***	**AG**	299 (32.6)	442 (29.2)	2765 (28.3)		
	**GG**	21 (2.3)	46 (3.0)	340 (3.5)		
**rs17655**	**CC**	239 (26.1)	354 (23.4)	2455 (24.6)	0.801	0.875
***ERCC5***	**GC**	435 (47.5)	737 (48.7)	4945 (49.6)		
	**GG**	242 (26.4)	425 (28.1)	2566 (25.8)		
**rs20417**	**CC**	857 (93.4)	1390 (91.6)	710 (91.1)	0.815	0.914
***PTGS2* (COX2)**	**GC**	60 (6.5)	125 (8.2)	69 (8.9)		
	**GG**	1 (0.1)	2 (0.1)	0 (0)		
**rs671**	**GG**	259 (28.1)	785 (52.1)	5372 (55.6)	**<0.001**	0.750
***ALDH2***	**GA**	642 (69.8)	603 (40.0)	3638 (37.7)		
	**AA**	19 (2.1)	119 (7.9)	648 (6.7)		
**rs2239751**	**AA**	417 (45.3)	709 (46.9)	2778 (44.6)	0.928	0.803
***CISH***	**CA**	394 (42.8)	653 (43.2)	2717 (46.6)		
	**CC**	109 (11.9)	165 (10.9)	731 (11.7)		
**rs243324**	**CC**	512 (55.7)	859 (56.7)	415 (53.5)	0.924	0.725
***SOCS1***	**TC**	348 (37.8)	571 (37.7)	305 (39.4)		
	**TT**	60 (6.5)	85 (5.6)	55 (7.1)		
**rs33932899**	**GG**	523 (57.0)	872 (57.7)	435 (56.0)	0.944	0.713
***SOCS1***	**CG**	341 (37.1)	562 (37.2)	287 (37.0)		
	**CC**	54 (5.9)	78 (5.2)	54 (7.0)		

^#^Results in current study. EC, esophageal cancer.

^&^Data from genome aggregation database (gnomAD) https://gnomad.broadinstitute.org/

*Data from Taiwan Biobank https://www.twbiobank.org.tw/new_web_en/index.php Bold values indicate statistical significance with a p value less than 0.05.

**Table 6:** Risk for esophageal cancer by ADH1B:rs1229984 and ALDH2: rs671.

Variables	genotype	Case	Control	Risk for esophageal cancer	
				OR (95% CI)	*p-value
**rs1229984**	**TT**	357 (39.1)	789 (52.2)	1	
***ADH1B***	**TC**	339 (37.1)	611 (40.4)	1.23 (1.02-1.47)	**0.028**
	**CC**	218 (23.9)	112 (7.4)	4.30 (3.32-5.58)	**<0.001**
**rs671**	**GG**	259 (28.2)	785 (51.9)	1	
***ALDH2***	**GA**	642(69.8)	603 (39.9)	3.23 (2.70-3.86)	**<0.001**
	**AA**	19 (2.1)	119 (7.9)	0.48 (0.29-0.80)	**0.005**

Bold values indicate statistical significance with a p value less than 0.05.

As for ALDH2:rs671, heterozygous GA (ALDH2*1/*2) was the most dominant genotype in EC patients, which they carried at significantly higher rates than the normal populations (69.8% vs. 39.9%); by contrast, wild-type GG (ALDH2*1/*1) was the dominant genotype among the normal population (28.2% vs. 51.9%, EC patients vs. the normal populations, **Table 5**). Among the normal Taiwanese population, those carrying the GA genotype had a 3.23-fold increased risk for EC compared to those carrying GG (GA/GG, crude OR[95% CI]=3.23 [2.70–3.86], *p*<0.001, **Table 6**). Consistent with previous meta-analysis results (31), a protective effect was found in rs671 homozygote AA (AA/GG, crude OR[95% CI]=0.48 [0.29–0.80], *p*=0.005, **Table 6**).

We then further analyzed the correlation between the candidate SNPs and the risk of MPC in EC patients. An increased risk of MPC was found in patients carrying the GA and AA genotypes of ALDH2:rs671 (*p*=0.021). Up to 22.0% and 31.6% of GA and AA carriers, respectively, had other malignancies in comparison to only 15% of GG carriers (**Table 7**). This correlation was more evident in patients who developed HNC, as more than 16% of GA carriers also had HNC (**Table 7**). The heterozygous genotype GC of ERCC5:rs17655 also correlated with increased risk of MPC (*p*=0.005), HNC (*p*=0.038), and gastrointestinal cancer (*p*=0.048). Moreover, CISH:rs2239751 was found to be significantly correlated with MPC in EC patients (*p*=0.033), with more than 29% of CC carriers also developing MPC. Notably, 7.3% of CC carriers had thoracic cancers, mostly lung cancer, which was a much higher incidence than in patients with the AA or CA genotypes (1.9% and 1.0%, respectively, *p*=0.002, **Table 7**). ADH1B:rs1229984 only displayed a borderline association with the risk of gastrointestinal cancer (*p*=0.085).

**Table 7:** Correlation between candidate SNPs and the incidence of MPC in EC patients.

SNP/Gene	Genotype	No.	MPC	*p*-value	Multiple MPC	*p*-value	HNC	*p*-value	GI Ca.	*p*-value	Thoracic Ca.	*p*-value
		920	184 (20.0)		28 (15.2)		128 (69.6)		39 (21.2)		20 (10.9)	
**rs1048943**	**TT**	508 (55.4)	98 (19.3)	0.609	13 (2.6)	0.515	66 (13.0)	0.704	22 (4.3)	**0.009**	10 (2.0)	0.869
**CYP1A1**	**CT**	348 (37.9)	71 (20.4)		11 (3.2)		52 (14.9)		10 (2.9)		9 (2.6)	
	**CC**	61 (6.7)	15 (24.6)		3 (4.9)		9 (14.8)		7 (11.5)		1 (1.6)	
**rs1229984**	**TT**	357 (39.1)	59 (16.5)	0.100	8 (2.2)	0.470	42 (11.8)	0.120	15 (4.2)	0.085	5 (1.4)	0.494
**ADH1B**	**TC**	339 (37.1)	72 (21.2)		13 (3.8)		46 (13.6)		19 (5.6)		9 (2.7)	
	**CC**	218 (23.9)	51 (23.4)		7 (3.2)		39 (17.9)		4 (1.8)		5 (2.3)	
**rs1695**	**AA**	596 (65.1)	122 (20.5)	0.750	19 (3.2)	1.000	87 (14.6)	0.465	24 (4.0)	0.941	11 (1.8)	0.306
**GSTP1**	**AG**	299 (32.6)	56 (18.7)		9 (3.0)		37 (12.4)		13 (4.3)		8 (2.7)	
	**GG**	21 (2.3)	5 (23.8)		0 (0)		4 (19.0)		0 (0)		1 (4.8)	
**rs17655**	**CC**	239 (26.1)	34 (14.2)	**0.005**	6 (2.5)	0.349	22 (9.2)	**0.038**	7 (2.9)	**0.048**	5 (2.1)	0.752
**ERCC5**	**GC**	435 (47.5)	106 (24.4)		17 (3.9)		71 (16.3)		26 (6.0)		11 (2.5)	
	**GG**	242 (26.4)	44 (18.2)		5 (2.1)		34 (14.0)		6 (2.5)		4 (1.7)	
**rs20417**	**CC**	857 (93.4)	177 (20.7)	0.252	26 (3.0)	1.000	123 (14.4)	0.242	39 (4.6)	0.142	17 (2.0)	0.156
***PTGS2***	**GC**	60 (6.5)	7 (11.7)		1 (1.7)		4 (6.7)		0 (0)		3 (5.0)	
**(COX-2)**	**GG**	1 (0.1)	0 (0)		0 (0)		0 (0)		0 (0)		0 (0)	
**rs671**	**GG**	259 (28.2)	38 (14.7)	**0.021**	3 (1.2)	0.079	22 (8.5)	**0.007**	10 (3.9)	0.766	5 (1.9)	0.486
**ALDH2**	**AG**	642 (69.8)	141 (22.0)		25 (3.9)		104 (16.2)		28 (4.4)		14 (2.2)	
	**AA**	19 (2.1)	6 (31.6)		0 (0)		2 (10.5)		1 (5.3)		1 (5.3)	
**rs2239751**	**AA**	417 (45.3)	76 (18.2)	**0.033**	15 (3.6)	0.683	54 (12.9)	0.131	13 (3.1)	0.289	8 (1.9)	**0.002**
**CISH**	**CA**	394 (42.8)	77 (19.5)		10 (2.5)		52 (13.2)		21 (5.3)		4 (1.0)	
	**CC**	109 (11.8)	32 (29.4)		3 (2.8)		22 (20.2)		5 (4.6)		8 (7.3)	
**rs243324**	**CC**	512 (55.7)	96 (18.8)	0.740	14 (2.7)	0.740	70 (13.7)	0.831	23 (4.5)	0.723	10 (2.0)	0.668
**SOCS1**	**TC**	348 (37.8)	78 (22.4)		12 (3.4)		51 (14.7)		15 (4.3)		8 (2.3)	
	**TT**	60 (6.5)	11 (18.3)		2 (3.3)		7 (11.7)		1 (1.7)		2 (3.3)	
**rs33932899**	**GG**	523 (57.0)	98 (18.7)	0.445	14 (2.7)	0.689	71 (13.6)	0.902	25 (4.8)	0.633	9 (1.7)	0.342
**SOCS1**	**CG**	341 (37.1)	76 (22.3)		12 (3.5)		50 (14.7)		13 (3.8)		9 (2.6)	
	**CC**	54 (5.9)	11 (20.4)		2 (3.7)		7 (13.0)		1 (1.9)		2 (3.7)	

MPC, multiple primary cancer; HNC, head and neck cancer; GI, gastrointestinal; Ca, cancer. Bold values indicate statistical significance with a p value less than 0.05.

In multivariate logistic analysis that adjusted for other potential variables, CISH:rs2239751_CC had strong significant correlation with increased risk of MPC and thoracic cancer (OR[95% CI]=1.99 [1.20–3.32], *p*=0.008 for MPC; OR[95% CI]=5.40 [1.83–15.91], *p*=0.002 for thoracic cancer, **Table 8**). Patients carrying the AA variant of ALDH2:rs671 had a 3.61-fold increased risk for MPC compared to wildtype GG carriers (OR[95% CI]=3.61[1.13–11.56], *p*=0.030, **Table 8**), whereas the AG genotype was significantly correlated with an increased HNC risk (OR[95% CI]=1.82 [1.08–3.07], *p*=0.030). Notably, AA carriers had a 7-fold increased risk of developing synchronous MPC (OR[95% CI]=7.55 [1.24–45.90], *p*=0.028, **Table 9**). Furthermore, ERCC5:rs17655_GC was correlated with a significantly greater risk of MPC and HNC than with CC (OR[95% CI]=2.15 [1.36–3.40], *p*=0.001 for MPC; OR[95% CI]=2.07 [1.21–3.55], *p*=0.008 for HNC, **Table 8**). The GG genotype was also significantly associated with the risk of developing HNC (OR[95% CI]=1.91 [1.05–3.49], *p*=0.035). Moreover, ERCC5:rs17655_GC was also significantly correlated with the risk of metachronous MPC before or after EC (*p*=0.011 and *p*=0.022, respectively, **Table 9**). Finally, ADH1B:rs1229984_CC was associated with MPC and HNC (*p*=0.117 and *p*=0.160, respectively).

**Table 8:** Risk SNPs for individual types of MPC analyzed by multivariate logistic regression.

Variables	genotype	No.	MPC		HNC		GI Ca.		Thoracic Ca.	
			adjusted OR (95% CI)	*p-value	adjusted OR (95% CI)	*p-value	adjusted OR (95% CI)	*p-value	adjusted OR (95% CI)	*p-value
**rs1048943**	**TT**	469	1		1		1		1	
***CYP1A1***	**CT**	318	1.10 (0.77-1.58)	0.593	1.27 (0.84-1.92)	0.256	0.65 (0.30-1.40)	0.266	1.17 (0.45-3.05)	0.744
	**CC**	54	1.39 (0.71-2.75)	0.339	1.53 (0.70-3.36)	0.291	2.21 (0.78-6.26)	0.136	0.81 (0.10-6.59)	0.841
**rs1229984**	**TT**	323	1		1		1		1	
***ADH1B***	**TC**	316	1.34 (0.90-2.00)	0.155	1.14 (0.71-1.83)	0.585	1.47 (0.70-3.06)	0.309	2.00 (0.65-6.16)	0.228
	**CC**	199	1.43 (0.91-2.25)	0.117	1.44 (0.87-2.39)	0.160	0.59 (0.19-1.87)	0.369	1.36 (0.35-5.32)	0.661
**rs17655**	**CC**	219	1		1		1		1	
***ERCC5***	**GC**	405	2.15 (1.36-3.40)	**0.001**	2.07 (1.21-3.55)	**0.008**	1.90 (0.80-4.55)	0.148	1.29 (0.40-4.18)	0.677
	**GG**	216	1.57 (0.93-2.65)	0.090	1.91 (1.05-3.49)	**0.035**	0.60 (0.19-1.97)	0.403	0.86 (0.21-3.52)	0.828
**rs671**	**GG**	228	1		1		1		1	
***ALDH2***	**AG**	599	1.47 (0.96-2.24)	0.074	1.82 (1.08-3.07)	**0.030**	1.11 (0.51-2.40)	0.794	1.50 (0.46-4.83)	0.501
	**AA**	17	3.61 (1.13-11.56)	**0.030**	2.75 (0.55-13.85)	0.219	1.07 (0.12-9.97)	0.952	4.87 (0.42-56.23)	0.204
**rs2239751**	**AA**	386	1		1		1		1	
***CISH***	**CA**	358	1.07 (0.74-1.55)	0.733	0.93 (0.60-1.43)	0.743	1.59 (0.76-3.32)	0.216	0.67 (0.19-2.36)	0.536
	**CC**	100	1.99 (1.20-3.32)	**0.008**	1.64 (0.92-2.94)	0.094	1.61 (0.55-4.74)	0.386	5.40 (1.83-15.91)	**0.002**

*adjusted for age, gender, tumor site, histology, chewing, drinking, and smoking.

MPC, multiple primary cancer; HNC, head and neck cancer; GI, gastrointestinal; Ca, cancer. Bold values indicate statistical significance with a p value less than 0.05.

**Table 9:** Risk SNPs for synchronous and metachronous MPC analyzed by multivariate logistic regression.

Variables	genotype	No	Risk for synchronous MPC (N=55)		Risk for metachronous MPC (SPC, N=55)		Risk for metachronous MPC (before EC, N=74)	
			adjusted OR (95% CI)	*p-value	adjusted OR (95% CI)	*p-value	adjusted OR (95% CI)	*p-value
**rs1048943**	**TT**	469	1		1		1	
***CYP1A1***	**CT**	318	0.80 (0.44-1.46)	0.472	1.12 (0.63-1.99)	0.694	1.36 (0.81-2.27)	0.243
	**CC**	54	1.07 (0.35-3.22)	0.909	1.20 (0.40-3.60)	0.747	1.44 (0.53-3.91)	0.471
**rs1229984**	**TT**	323	1		1		1	
***ADH1B***	**TC**	316	1.00 (0.51-1.95)	0.999	1.72 (0.88-3.37)	0.114	1.26 (0.71-2.22)	0.426
	**CC**	199	1.23 (0.60-2.54)	0.573	1.85 (0.89-3.87)	0.102	1.05 (0.54-2.03)	0.896
**rs17655**	**CC**	219	1		1		1	
***ERCC5***	**GC**	405	1.24 (0.61-2.51)	0.551	2.42 (1.14-5.16)	**0.022**	2.61 (1.24-5.48)	**0.011**
	**GG**	216	1.07 (0.47-2.43)	0.873	1.12 (0.44-2.84)	0.806	2.63 (1.18-5.86)	**0.018**
**rs671**	**GG**	228	1		1		1	
***ALDH2***	**GA**	599	2.09 (0.95-4.59)	0.068	1.52 (0.74-3.13)	0.253	1.00 (0.57-1.77)	0.992
	**AA**	17	7.55 (1.24-45.90)	**0.028**	4.30 (0.80-23.19)	0.090	0.98 (0.12-8.29)	0.984
**rs2239751**	**AA**	386	1		1		1	
***CISH***	**CA**	358	1.67 (0.87-3.18)	0.122	0.72 (0.39-1.33)	0.291	0.91 (0.53-1.54)	0.712
	**CC**	100	3.25 (1.49-7.11)	**0.003**	1.46 (0.67-3.16)	0.342	1.31 (0.62-2.74)	0.480

*adjusted for age, gender, tumor site, histology, chewing, drinking, and smoking.

MPC, multiple primary cancer; HNC, head and neck cancer; SPC, second primarycancer; EC, esophageal cancer. Bold values indicate statistical significance with a p value less than 0.05.

We defined CISH:rs2239751_CC, ALDH2:rs671_AG/AA, ERCC5:rs17655_GC/GG, and ADH1B: rs1229984_CC as risk genotypes for MPC in EC patients. According to multivariate analysis, patients carrying these four risk genotypes had more than 40-fold increased risk of MPC compared to those without (OR[95% CI]=40.25 [6.77–239.50], *p*<0.001, **Table 10**). The cumulative effect of risk SNPs was also significant in the risk of having HNC and the risk of developing SPC after EC (OR[95% CI]=9.75 [1.87–50.84], *p*=0.007 for HNC; OR[95% CI]=12.57 [1.14–138.8], *p*=0.039 for SPC, **Table 10**).

**Table 10:** Cumulative effects of risk SNPs for MPC.

Risk genotype No.	No.	Risk for MPC		Risk for HNC		Risk for metachronous MPC (SPC)		Risk for metachronous MPC (before EC)	
		adjusted OR (95% CI)	*p-value	adjusted OR (95% CI)	*p-value	adjusted OR (95% CI)	*p-value	adjusted HR (95% CI)	*p-value
**0**	41	1		1		1		1	
**1**	248	2.37 (0.69-8.14)	0.170	1.33 (0.37-4.76)	0.666	1.29 (0.16-10.64)	0.816	1.62 (0.36-7.32)	0.532
**2**	376	3.13 (0.93-10.50)	0.065	2.08 (0.60-7.18)	0.248	3.43 (0.45-26.01)	0.232	1.59 (0.36-7.00)	0.544
**3**	156	4.05 (1.17-14.03)	**0.027**	3.27 (0.92-11.62)	0.068	2.89 (0.36-23.22)	0.318	2.32 (0.50-10.67)	0.282
**4**	13	40.25 (6.77-239.50)	**<0.001**	9.75 (1.87-50.84)	**0.007**	12.57 (1.14-138.8)	**0.039**	4.76 (0.64-35.48)	0.128

*adjusted for age, gender, tumor site, histology, chewing, drinking, and smoking.

Risk genotypes: CISH: rs2239751_CC; ALDH2:rs671_AG/AA; ADH1B: rs1229984_CC, ERCC5:rs17655_ GC/GG.

MPC, multiple primary cancer; SPC, second primary cancer; EC, esophageal cancer. Bold values indicate statistical significance with a p value less than 0.05.

**Figure 2:**
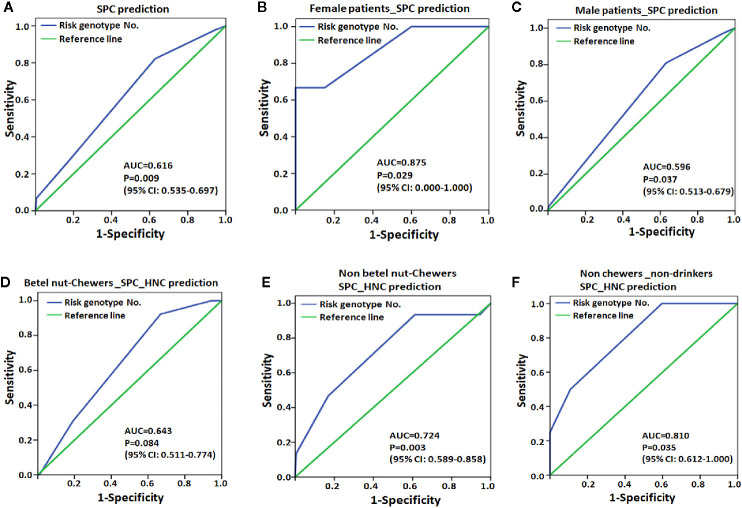
Receiver operating characteristic (ROC) plot for MPC risk genotype was used to differentiate the EC patients with second primary cancer (SPC, A-C) or with HNC after EC (SPC_HNC, D-F) from those without. **(A)** EC patients. **(B)** Female EC patients. **(C)** Male patients. **(D)** Patients with or without **(E)** the habit of chewing betel nut. **(F)** Patients without the habit of chewing betel nut and drinking alcohol. AUC, area under the receiver operating characteristic curve; CI, confidence interval.

The ROC curve further revealed that the number of risk genotypes had an excellent capability for SPC in female patients (AUC=0.875, **Figure 2B**) but poor capability in all patients as well as in male patients (**Figures 2A, C**, AUC=0.616 and 0.596, respectively). For the development of HNC after EC (SPC_HNC), the cumulating risk genotypes had a better capability in non-chewers (AUC=0.724, **Figure 2E**) compared to betel nut chewers (AUC=0.643, **Figure 2D**). Notably, the risk genotypes had an excellent capability for SPC_HNC in patients with no chewing and drinking habits (AUC=0.810, **Figure 2F**).

## Discussion

Our study is the first to systematically investigate risk factors and predictive biomarkers for MPC in EC. Based on our results, age was a risk factor for MPC. Head and neck cancers, such as oral cancer, are most prevalent between 40 and 60 years of age, whereas cancers in the digestive system, such as gastric cancer and colon cancer, are most prevalent among elderly people aged >70 years. Our results reveal that younger patients with EC have an increased risk of having HNC and that older patients have a higher probability of developing gastrointestinal cancers (**Table 3**). Moreover, our results showed that betel chewing is the most predominant unfavorable habit correlated with the incidence of MPC in EC, especially in patients with HNC (**Table 3**). Both cigarette-smoking and alcohol-drinking were obviously correlated with an increased incidence of MPC according to Chi-square analysis (**Table 2**), but no significant effect was observed in the multivariate regression model adjusted for other variables (**Table 3**). Over 80% of these EC patients have tobacco (N=733) and alcohol (N=703) consumption, and about 35% of patients (N=299) have betel nut chewing behavior. Since all of these betel nut chewers also had at least one of the two other unfavorable habits, we suggest that the cumulative effect of these dangerous habits is crucial in the incidence of MPC in EC.

ALDH2:rs671 and ADH1B:rs1229984 have been frequently demonstrated to strongly correlate with the risk of EC (11–16). The genotype distribution of ADH1B:rs1229984 also showed no significant difference between the normal Taiwanese and whole East Asian populations (*p*=0.905); by contrast, there was a significant difference between the EC subjects and the normal Taiwanese population (*p*<0.001, **Table 5**). The percentage of ALDH2 deficiency in Taiwan has been ranked number 1 globally, with around 48% of Taiwanese people carrying the variant allele; however, this did not have a statistically significant difference when compared to the whole East-Asian population, according to our analysis (*p*=0.750). Moreover, the genotype distribution of rs671 was significantly different between our EC subjects and the normal Taiwanese population (*p*<0.001). Up to approximately 70% of EC subjects carry the GA variant. We further demonstrated that GA carriers had an increased risk of developing HNC (**Table 8**) and synchronous MPC (**Table 9**). EC patients carrying the null variant AA also had a significant risk for MPC, especially for synchronous MPC (**Table 9**). Although alcohol is generally considered to be metabolized in the liver, some studies provide evidence to support the hypothesis that the exposure of alcohol-derived acetaldehyde may occur in the oral cavity since high salivary acetaldehyde was found in ALDH2-deficient subjects after drinking alcohol (32, 33). The protective role of ALDH2 against DNA damage induced by acetaldehyde in the esophageal squamous epithelium has also been reported (34). Whether the genetic effect of ALDH2_rs671 on the development of EC and HNC is mediated by regulating the local carcinogen action of acetaldehyde needs to be clarified by further research.

ERCC5, a single-stranded structure-specific DNA endonuclease, plays an essential role in the nucleotide excision repair machinery. rs17655 is a non-synonymous SNP in the coding region of ERCC5 and causes a 1104 amino acid change from Asp to His (Asp1104His). In our results, rs17655 was not associated with the risk of EC (**Table 5**). However, heterozygote GC carriers had a significantly increased risk for developing HNC and metachronous MPC (**Tables 8** and **9**). A previous study revealed that the rs17655 heterozygote carriers exhibited an increased risk of laryngeal cancer among heavy smokers (35). Thus, the function of rs17655 in MPC of EC patients is possibly due to its impaired repair function in response to environmental toxins, which leads to the development of HNC.

We found the novel biomarker CISH:rs2239751 to be significantly associated with MPC in EC patients, especially in combination with other thoracic cancers, particularly lung cancer (**Tables 7** and **8**). CISH belongs to the family of SOCS proteins, one of the key mechanisms regulating signaling derived from cytokines and growth factors, and plays important anti-inflammatory and tumor-suppressive roles (36). Degradation of receptors or associated proteins is one of the mechanisms by which SOCS proteins negatively regulate cytokine signaling or growth factors. CISH has been known to negatively regulate pathways induced by GH, IL-2, IL-3, IL-5, GM-CSF, EPO, and PRL (36). CISH:rs2239751 is a 5’UTR variant in transcript variant-1, which is reportedly correlated with persistent HBV infection (37). The minor allele C has also been found to be associated with susceptibility to tuberculosis in the Chinese Han population (38). The minor allele frequency of rs2239751 among the global population is only about 0.0914 (https://doi.org/10.1101/531210) This frequency also dramatically increases in the East Asia population to about 0.3356, which is close to the minor allele frequency in our population of EC patients at 0.3330. We also found that patients carrying CC had >5 odds of also having lung cancer (**Table 9**). Whether CISH:rs2239751 is also correlated with the incidence of lung cancer is worthy of future investigation.

We analyzed the cumulative effect of these MPC risk genotypes and revealed that patients carrying all 4 risk genotypes had over 40-fold and 12-fold increased risks of having MPC and SPC, respectively (**Table 10**). Although only 1.4% (13 out of 920) of the EC subjects carried 4 risk genotypes, it is a considerable number among cases of esophageal cancer globally (over 500,000/per year, new cases). Furthermore, the ROC curve analysis revealed that the risk genotype had an excellent capability for SPC in the low-risk population, including female patients (AUC=0.875) and those without drinking and chewing habits (AUC=0.810, **Figure 2**). It is reasonable that the genetic effects were more evident in patients without exposure to unfavorable lifestyle factors since these habit-related human carcinogens greatly impact cancer development and, therefore, probably masked the genetic effects for SPC.

Taken together, the study demonstrated for the first time that a set of risk SNPs, ALDH2:rs671, CISH:rs2239751, ERCC5:rs17655, and ADH1B:rs1229984, have great potential in predicting the incidence of MPC in EC. Genetic testing for these SNP variants would be beneficial for the early diagnosis of SPC. The limitations of the study were as follows: 1) there was no validation cohort, and 2) the lack of clear information to separate ever users and current users based on the use of tobacco, alcohol, and betel nut accurately.

## Data Availability Statement

The datasets presented in this study can be found in online repositories. The names of the repository/repositories and accession number(s) can be found below: European Nucleotide Archive, PRJEB41367.

## Ethics Statement

The studies involving human participants were reviewed and approved by The study was approved by the ethical committee of National Taiwan University Hospital (NTUH, 201803015RIND). The patients/participants provided their written informed consent to participate in this study.

## Author Contributions

P-WY, M-CL, M-HT, EC, P-JL, and J-ML provided the concept and design of the study. P-WY and M-CL performed the literature search and analyzed the data. P-WY, M-CL, and J-ML wrote the manuscript. EC, P-JL, and J-ML revised the manuscript. P-WY and M-CL performed the experiments. P-JL, C-PW, T-CC, C-NN, JC, M-SH, P-JL, and J-ML provided clinical research resources. All authors contributed to the article and approved the submitted version.

## Funding

This study was supported by the Ministry of Science and Technology (MOST 107-2314-B-002-148, MOST 108-2314-B-002-017, MOST 109-2314-B-002-273, MOST 107-2314-B-002-248-MY3, and MOST 109-0210-01-18-02), the National Taiwan University Hospital (NTUH.108-S4280), and the Ministry of Health and Welfare, Taiwan (MOHW108-TDU-B -211-124017 and MOHW109-TDU-B-211-134017).

## Conflict of Interest

The authors declare that the research was conducted in the absence of any commercial or financial relationships that could be construed as a potential conflict of interest.

## Abbreviations

EC, Esophageal cancer; MPCs, Multiple primary cancers; SNPs, Single-nucleotide polymorphisms; HNC, Head and neck cancer; GI ca., Gastrointestinal cancer; SPC, Second primary cancer.
